# A systematic review and meta‐analysis: Assessment of hospital walking programs among older patients

**DOI:** 10.1002/nop2.1496

**Published:** 2022-11-28

**Authors:** Christine Loyd, Yue Zhang, Tara Weisberg, James Boyett, Elizabeth R. Huckaby, Jeri Grundhoefer, Steve Otero, Lisa Roberts, Samantha Giordano‐Mooga, Carmen Capo‐Lugo, Catherine H. Smith, Richard E. Kennedy, Barbara J. King, Cynthia J. Brown

**Affiliations:** ^1^ Division of Gerontology, Geriatrics, and Palliative Care, Department of Medicine, UAB School of Medicine University of Alabama Alabama Birmingham USA; ^2^ Department of Clinical and Diagnostic Sciences, UAB School of Health Professions University of Alabama Alabama Birmingham USA; ^3^ Department of Physical Therapy, UAB School of Health Professions University of Alabama Alabama Birmingham USA; ^4^ Lister Hill Library of the Health Sciences University of Alabama at Birmingham Alabama Birmingham USA; ^5^ School of Nursing University of Wisconsin Wisconsin Madison USA; ^6^ Department of Medicine Louisiana State University Health Sciences Center Louisiana New Orleans USA

**Keywords:** hospitalization, meta‐analysis, mobility, nursing care, older adults, systematic review

## Abstract

**Aim:**

The aim of this study is to assess effect of hospital walking programs on outcomes for older inpatients and to characterize hospital walking dose reported across studies.

**Design:**

A systematic review and meta‐analysis examining impact of hospital walking and/or reported walking dose among medical‐surgical inpatients. For inclusion, studies were observational or experimental, published in English, enrolled inpatients aged ≥ 65 yrs hospitalized for medical or surgical reasons.

**Methods:**

Searches of PubMed, CINAHL, Embase, Scopus, NICHSR, OneSearch, ClinicalTrials.gov, and PsycINFO were completed in December 2020. Two reviewers screened sources, extracted data, and performed quality bias appraisal.

**Results:**

Hospital walking dose was reported in 6 studies and commonly as steps/24 hr. Length of stay (LOS) was a common outcome reported. Difference in combined mean LOS between walking and control groups was −5.89 days. Heterogeneity across studies was considerable (*I* ^*2*^ = 96%) suggesting poor precision of estimates. Additional, high‐quality trials examining hospital walking and patient outcomes of older patients is needed.

## 1 INTRODUCTION

Low hospital mobility is defined as movement limited to bed or bed to chair transfers with little walking during hospitalization (Wald et al., 2019). Low mobility among hospitalized older patients is a very common occurrence (Brown et al., 2004; Brown et al., 2009; Fisher et al., 2011; Zisberg et al., 2011), and in the context of age‐related loss of muscle mass and strength, accelerates further loss leading to decline in functional independence, new institutionalization, and death (Brown et al., 2004; Brown et al., 2016; Gill et al., 2004; Gill et al., 2010; Hirsch et al., 1990). Despite abundant evidence supporting the need to move during hospitalization, mobility and mobility assessment among older inpatients is not implemented broadly across healthcare systems (Wald et al., 2019). Identifying the combined effect of hospital walking on outcomes of older patients could present new evidence in support of its adoption as standard practice.

### 1.1 Background

The negative impact of low hospital mobility for older patients has encouraged the development of programs for stimulating walking activity among this population. These hospital‐based interventions are commonly led by nurses, physical therapists, physicians, and other healthcare professionals as part of a collaborative multi‐disciplinary team; and often include progressive mobilization, a process whereby patients progress from sitting to standing to stepping in place to walking on the unit (Wald et al., 2019). Some interventions include walking as part of a multicomponent program. For example, an intervention aimed at preventing delirium during hospitalization includes an orientation component, a cognitive activities component, a sleep component, and a mobility component (Inouye et al., 1999). Another similar intervention protocol includes orienting communication, oral and nutritional assistance, and early mobilization following surgery (Chen et al., 2017). Alternatively, other hospital‐based programs focus solely on getting adult patients out of bed and walking (Brown et al., 2016; Kakutani et al., 2019; Valenzuela et al., 2020).

Promotion of programs for hospital walking has centred on beneficial change in key clinical outcomes; in particular, outcomes related to the patient or to healthcare utilization. As described in a scoping review of hospital‐based mobility studies in adult patients aged ≥ 35 years, a variety of outcomes have been measured including hospital length of stay (LOS), functional status (e.g. activities of daily living, ADL, independence), walking amount and capacity, in‐hospital physical therapy receipt, hospital costs, mobility tests such as gait speed and Timed Up and Go tests, falls in the hospital, 30‐day emergency department visits and 30‐day hospital readmissions (Smart et al., 2018). Generally, studies have reported that hospital‐based mobility programs are useful for older patients by preventing declines in ADL and community mobility, reducing hospital LOS, and reducing discharge to a long‐term care facility (Brown et al., 2016; Cohen et al., 2019; Hastings et al., 2014). Yet, the benefits are not absolute across studies—some studies show conflicting results for the same outcome. For example, a 2015 quasi‐experimental study in Israel showed that a hospital walking program reduced odds of ADL decline while a 2010 randomized controlled trial in the USA found no such benefit (Brown et al., 2016; Cohen et al., 2019). Similarly, one mobility study reported a reduced hospital LOS among older adults in the intervention group while another claimed to not have a similar effect (Chen et al., 2017; Wnuk et al., 2016). Clarity on the impact of hospital walking for older patients could encourage adoption of hospital walking as routine practice more broadly. To the best of our knowledge, combined effects analysis of outcomes following hospital walking among older inpatients has not been completed.

## 2 THE REVIEW

### 2.1 Aims

This systematic review and meta‐analysis was designed to identify and consider all published studies examining hospital walking among older inpatients. A systematic review was conducted to collate existing evidence related to hospital walking interventions and outcome measures (length of stay) based on pre‐specified inclusion criteria to minimize risk of bias (Higgins, Morgan, et al., 2022; Higgins, Thomas, et al., 2022). The meta‐analysis allowed us to combine results from identified studies to evaluate the impact of walking interventions on patient outcomes. The primary aim of this investigation was to determine whether hospital walking improves outcomes among inpatients ≥65 years. Additionally, in light of an increase in the development and use of objective measures of mobility such as accelerometers (Smart et al., 2018), the secondary aim of this study was to characterize hospital walking dose in steps or distance units reported across studies for older inpatients.

## 3 METHODS

### 3.1 Design

A systematic review and meta‐analysis was completed. Preferred Reporting Items for Systematic Reviews and Meta‐Analysis (PRISMA) guidelines were followed for study design and reporting study findings (Moher et al., 2009). The study protocol is registered with PROSPERO International prospective register of systematic reviews (CRD42018112786). Patient consent was not needed for this study.

### 3.2 Search methods

With the help of an experienced librarian (CHS), electronic database searches of PubMed CINAHL, Embase, Scopus, NICHSR OneSearch, ClinicalTrials.gov, and PsycINFO were first executed in October 2018 and updated in December 2020. The search strategy was based on subject headings and keywords related to hospital walking interventions, hospitalized older adults, and recovery concepts. The terms used for the PubMed search are given in Table S1. The reference lists of included articles were also reviewed to identify eligible studies not captured in our search to improve the integrity of the study. The source search was not limited to a specified time‐period. Eligible studies enrolled participants that were ≥ 65 years and hospitalized in an inpatient facility for surgical and/or acute medical reasons and used a walking intervention during hospitalization or quantified walking dose in steps or distance units in the hospital. Interventions that included other components (including exercise‐related activities) were acceptable if walking was the major exercise component in the intervention. If other types of exercise were involved in an intervention and walking was not the main component, then the intervention study was excluded. Additionally, eligible studies included those published in English and were either observational (prospective, retrospective) or experimental (randomized controlled trials, quasi‐experimental) in design. Studies completed in other clinical settings including intensive care units, subacute care units or rehabilitation hospitals, nursing homes and other long‐term care facilities, or in outpatient clinics were excluded. Study authors were contacted if additional information or clarification was needed to assess eligibility. Eligibility was not determined by which clinical endpoints were examined in a study, but the research team hypothesized a priori that identified studies would focus on walking‐induced change in functional outcomes, other health‐related outcomes, and healthcare utilization.

### 3.3 Search outcome

Using pre‐specified eligibility criteria, two independent reviewers screened abstracts identified through the database searches and a third reviewer resolved decision conflicts to establish consensus. Prior to full‐text review, a quality control process was implemented whereby the study statistician (YZ) randomized 20% of excluded abstracts for re‐review. No additional abstracts were reclassified into the include category. Full‐texts of included studies were located and evaluated for eligibility in a similar fashion by two independent reviewers and a third that resolved conflicts. To expedite and organize the reviewing process, an online software (Covidence) was used.

### 3.4 Quality appraisal

The reviewers that extracted data also completed quality bias analysis for each included study. Two independent reviewers assessed quality bias and a third reviewer resolved decision conflicts. The observational studies included in the review were assessed for bias using the Risk of Bias In Non‐randomized Studies–of Exposure (ROBINS‐E) tool (Higgins, Morgan, et al., 2022; Higgins, Thomas, et al., 2022). The Risk of Bias in Non‐randomized Studies–of Interventions (ROBINS‐I) tool was used to assess bias among included interventions that were not randomized trials (Sterne et al., 2016). The Jadad scale for reporting randomized controlled trials and the Revised Cochrane risk‐of‐bias tool for randomized trials 2 (RoB2) were used to assess bias in the included randomized intervention studies (Jadad et al., 1996; Sterne et al., 2019). Domains of risk of bias were variable based on the study design which is illustrated in Table S2 and Figures S1 and S2. No studies were excluded from this review due to quality bias assessment findings because they met the inclusion criteria for this review.

### 3.5 Data abstraction

Two independent reviewers extracted study characteristics and design, hospital setting, study population, interventions used, and outcomes reported (including time points) from included sources. Data was stored in individual Microsoft Excel database files and examined for accuracy by a third reviewer. Data extracted was combined into a single database and relevant information was used to create Table 1. Table 1 illustrates all included studies organized by year published. General information about the study (geographical location, time‐frame, design), about the study sample (groups, sample size, mean age), and steps or distance walked/time during hospitalization are present. Additionally, hospital LOS data (which was used in meta‐analysis) and hospital walking distance or steps were also included in Table 1.

**TABLE 1:** Characteristics of included studies

Author (year published)	Study period, country	Study design	Groups	Sample size (no.)	Age in years (mean ± SD)	Hospital setting	Hospital walking dose	Hospital LOS (mean days ± SD)
Yohannes and Connolly (2003)	NR, United Kingdom (UK)	Quasi‐experimental	Intervention (GFSO, GFSA, RSO, RSA), No Control	Total = 120, GFSO = 30, GFSA = 30, RSO = 30, RSA = 30	GFSO = 75 ± 7, GFSA = 75 ± 7, RSO = 74 ± 8, RSA = 74 ± 7	Acute Care	NR	GFSO = 11.7 ± 12.1; GFSA = 10.04 ± 11.4RSO = 9.96 ± 12.5; RSA = 8.89 ± 12.5
Killey and Watt (2006)	NR, Australia	Intervention, not otherwise specified	Intervention (IG), Control	Total = 55, IG = 27, Control = 28	IG = 84.00 ± 6.19, Control = 82.54 ± 7.45	Acute Care	Mean meter/24 hr: IG: Initial = 38.64 ± 27.13, Final: 79.44 ± 58.03, Control: initial = 32.11 ± 32.83, Final = 47.86 ± 47.7	NR
Stenvall et al. (2012)^a^	2000–02, Sweden	RCT	Intervention (IG), Control	Total = 64, IG = 28, Control = 36	IG = 81.0 ± 5.8, Control = 83.4 ± 6.4	Acute Care	NR	IG = 20.0 ± 12.0, Control = 32.3 ± 35.3
Fisher et al. (2011)	2009, USA	Observational	Grouped by mean daily steps during hospitalization	Total = 239	All = 76.6 ± 7.6	Acute Care	Mean steps/24 hr: 739.7 (Q1‐Q3: 89–1,014)	Low (359.8 steps) = ≥7, Medium (689.6 steps) = 5–6, High (882.9 steps) = 3–4
Adogwa et al. (2017)	2010–11, USA	Observational	Grouped by time to ambulation: Early, Late, No Control	Total = 125, Early = 66, Late = 59	Early = 73.72 ± 6.2, Late = 73.22 ± 5.31	Surgery	Mean Ft/1st walking day: Early = 116.34 ± 133.27, Control = 75.84 ± 94.14 Mean ft/discharge day: Early = 245.23 ± 270.75, Control = 132.20 ± 164.83	Early = 5.33 ± 3.0, Late = 8.11 ± 7.70
Hastings et al. (2014)^a^	2012, USA	Intervention, not otherwise specified	STRIDE (intervention), Control	Total = 128, STRIDE = 92, Control = 35	Median Age: STRIDE = 74 (IQR: 66–80), Control = 75 (IQR: 67–83)	Acute Care	NR	Median: STRIDE = 4.7 (Q1‐Q3: 2.8–8.9), Control = 5.7 (Q1‐Q3: 3.0–8.5)
Izawa et al. (2015)	2006–12, Japan	Observational	Grouped by gender: Men, Women	Total = 268	Men = 73.4 ± 6.2, Women = 73.1 ± 5.7	Acute Cardiac Care	Mean steps/24 hr: Men = 4,037.33 ± 1866.81, Women = 2,651.35 ± 1889.92	Men = 26.9 ± 15.4, Women = 27.6 ± 13.8
Fisher et al. (2016)^a^	2010–11, USA	Observational	Grouped by re‐hospitalization: Re‐hospitalized (Control), Not re‐hospitalized	Total = 164, Rehosp. = 26, Not = 138	All = 76.25 ± 7.02, Rehosp. = 79.2 ± 7.99, Not = 75.67 ± 6.67	Acute Care	Mean steps/24 hr: All = 626 (Q1‐Q3: 266–1,403), Rehosp. = 323 (Q1‐Q3: 71–652), Not = 674 (Q1‐Q3: 324–1,604)	Rehosp. = 6.46 ˃ ±4.96, Not = 4.99 ± 4.13
Brown et al. (2016)^a^	2010–11, USA	RCT	Mobility Program (MP), Usual Care (UC, Control)	Total = 100, MP = 50, UC = 50	All = 73.9 ± 6.96, MP = 74.4 ± 6.9, UC = 73.4 ± 7.0	Acute Care	NR	MP = 4.6 ± 4.0, UC = 3.6 ± 2.4, Median = 3
Wnuk et al. (2016)^a^	NR, Poland	RCT	Intervention (Exp1 [backwards], Exp2 [forwards]), Control	Total = 47, Exp1 = 15, Exp2 = 16, Control = 16	Exp1 = 68 ± 3, Exp2 = 70 ± 3, Control = 69 ± 4	Surgery	NR	Exp1 = 5.87 ± 0.83; Exp2 = 6.5 ± 0.63; Control = 6.56 ± 0.73
Bürge et al. (2017)	NR, Switzerland and Belgium	RCT	Experimental (EG), Control	Total = 160, EG = 78, Control = 82	EG = 81.7 ± 7.7, Control = 81.7 ± 7.7	Acute Psychogeri‐atric Care	NR	NR
Chen et al. (2017)	2009–12, Taiwan	RCT	mHelp (intervention), Control	Total = 377, mHELP = 197, Control = 180	mHELP = 74.3 ± 5.8, Control = 74.8 ± 6.0	Surgery	NR	Median: mHELP = 12.0 (IQR: 6), Control = 14.0 (IQR:9)
Cohen et al. (2019)^a^	2015–16, Israel	Quasi‐experimental	Intervention (IG), Control	Total = 377, IG = 188, Control = 189	All = 75.4 ± 7.0, IG = 75.4 ± 7.3, Control = 75.3 ± 6.8	Acute Care	Mean steps/24 hr: IG = 3,205, Control = 1791, *p* < .001	IG = 5.8 ± 3.0, Control = 6.5 ± 4.3
Kakutani et al. (2019)^a^	2009–15, Japan	Quasi‐experimental	Intervention (IG), Control	Total = 136, IG = 75, Control = 61	IG = 79 ± 11, Control = 81 ± 11	Acute Cardiac Care	NR	IG = 33 ± 25; Control = 51 ± 36
Ortiz‐Alonso et al. (2020)^b^	2012–14, Spain	RCT	Intervention (IG), Control	Total = 268, IG = 143, Control = 125	IG = 88 ± 5, Control = 88 ± 5	Acute Care	NR	Median: IG = 6 (IQR:4) Control = 7 (IQR:5)
Valenzuela et al. (2020)^b^	2012–14, Spain	RCT	Intervention: 3 subgroups—Responders, Non‐responders (Non), Adverse responders (Adverse); Control	Total = 268, IG = 143 (Responders = 69, Non = 60, Adverse = 14), Control = 125	Each IG subgroup = 88 ± 5, Age range = 75–102, Control = NR	Acute Care	NR	IG: Responders = 6 ± 4.5, Non = 6 ± 3, Adverse = 8 ± 7.5, Median, All IG = 7 (IQR:4) Control = NR

Abbreviations: GFSA, gutter frame with supplemental air; GFSO, gutter frame with supplemental oxygen; LOS, length of stay; NA, not applicable; NR, not reported; RCT, randomized controlled trial; RSA, rollator with supplemental air; RSO, rollator with supplemental oxygen.

^a^ Denotes studies that could be used in meta‐analysis for hospital length of stay.

^b^ Denotes two unique publications using the study sample. Will refer to these two publications as the same study in the main text.

### 3.6 Synthesis

Hospital LOS data (Table 1) was used to calculate effect sizes for each study to allow comparison across studies. The difference between mean LOS between the walking and control groups for each study was calculated and combined using random effects meta‐analysis to determine the overall effect of increased hospital walking on LOS. If a study reported LOS as median and interquartile range (IQR, reported as values for Q1 and Q3), the mean and standard deviation were estimated using a previously reported methodology (Wan et al., 2014). The 95% confidence intervals for each study and the combined effect were also calculated. The random effects model was used to account for heterogeneity between studies (Hedges & Vevea, 1998), which was assessed using Cochran's Q (Hedges & Olkin, 1985). Additionally, the heterogeneity was assessed with the *I* ^2^ statistic analysis. Studies were weighted by sample size to limit undue effects of smaller studies and funnel plots were used to assess study bias and the robustness of findings (Rosenberg, 2005; Sterne et al., 2011). Meta‐analysis was conducted using the *metafor package* in R Studio (Auckland, New Zealand; Viechtbauer, 2010).

## 4 RESULTS

**FIGURE 1:**
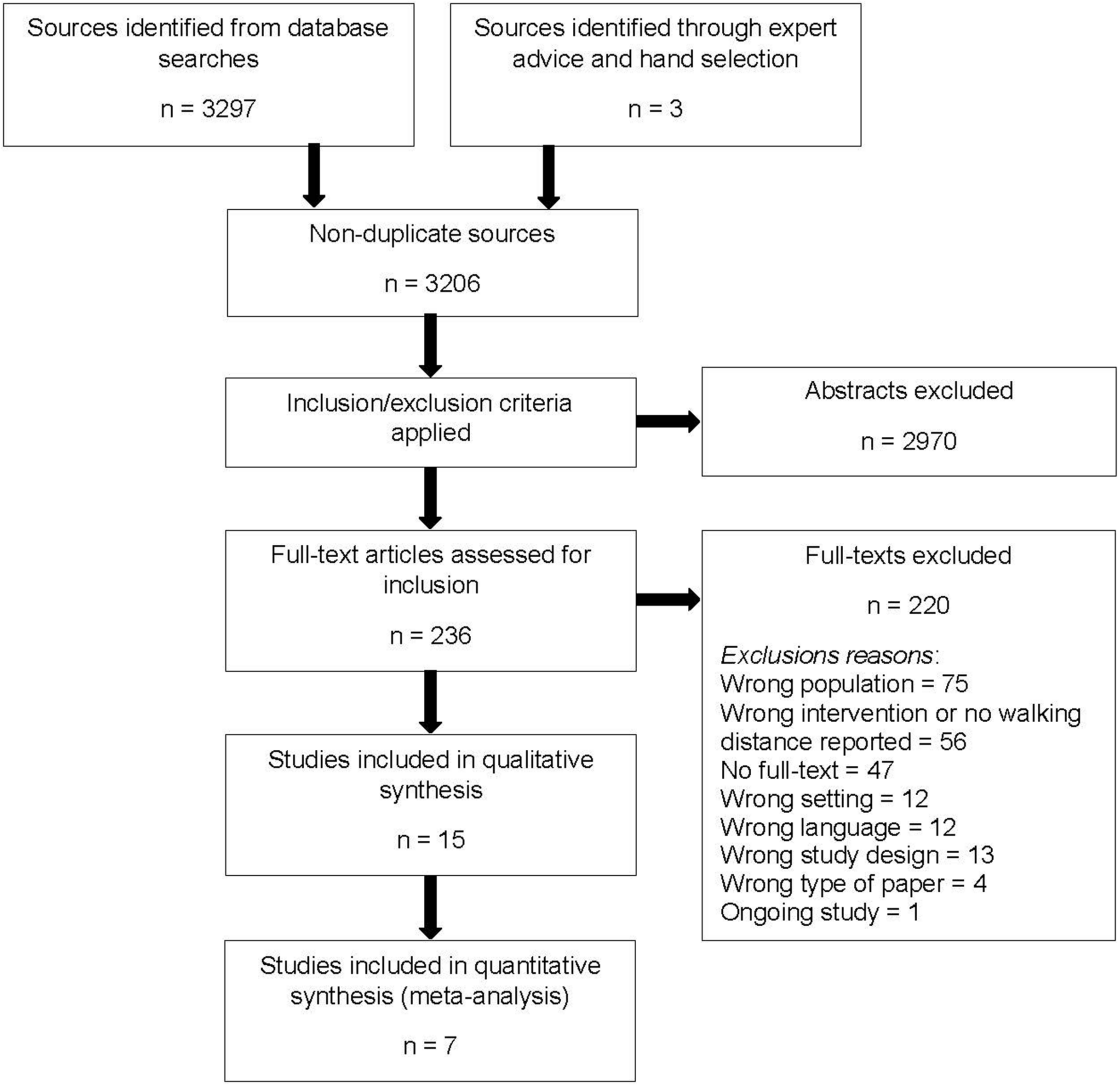
PRISMA diagram

The initial and updated database searches yielded 3,297 study titles and abstracts. Duplicate titles/abstracts were removed and 3,206 non‐duplicate sources were assessed for eligibility. Of these, 236 study full‐texts were reviewed and 16 publications met eligibility criteria. Two publications were completed on the same study sample (Ortiz‐Alonso et al., 2020; Valenzuela et al., 2020) resulting in 15 unique studies included in the analysis. Of the included studies, 13 reported hospital LOS but only 7 studies reported findings that could be used in meta‐analysis (Figure 1). Studies that could not be included in meta‐analysis did not include a control group (Adogwa et al., 2017; Fisher et al., 2011; Izawa et al., 2015; Yohannes & Connolly, 2003) or reported LOS data that could not be used to calculate mean and standard deviation and thus could not be added to the combined effects analysis (Chen et al., 2017; Ortiz‐Alonso et al., 2020).

Table 1 shows general characteristics of included studies. Most studies were conducted during the years of 2000 to 2016; 4 studies did not report the study period. Five studies were completed in European hospitals (UK, Sweden, Spain, Switzerland, Belgium, and Poland), 5 in American hospitals, 2 in Japanese hospitals, 1 in a Taiwanese hospital, 1 in an Israeli hospital, and 1 in an Australian hospital. The majority of studies, 11/15 (73%), occurred on acute care units with medical patients while 3 studies used surgical patients only. One study occurred among patients with dementia in an acute psychiatric setting. Eleven studies were interventions (Ortiz‐Alonzo et al. and Valenzuela et al. are counted as a single study), 5 of which were described as quasi‐experimental or as interventions but not otherwise specified, and 7 were randomized controlled trials (RCT). Interestingly, only 1 RCT was completed in an American hospital. Sample sizes across studies ranged from 47 to 377 patients with a total sample size of 2,628. All patients included in this systematic review were at least 65 years of age. One study reported recruiting patients ≥60 years, however the entire study sample only enrolled patients ≥65 years (Yohannes & Connolly, 2003). Hospital LOS was the most commonly reported outcome across studies, but additional outcomes were also reported. Discharge destination (Adogwa et al., 2017; Hastings et al., 2014; Kakutani et al., 2019), 30‐day readmission (Hastings et al., 2014; Kakutani et al., 2019), and changes in ADL (Brown et al., 2016; Bürge et al., 2017; Cohen et al., 2019; Killey & Watt, 2006; Ortiz‐Alonso et al., 2020; Stenvall et al., 2012) were measured occasionally. While changes in ADL score were reported across multiple studies, there was considerable variability in data collection time points and in how the data were reported hindering combined effect analysis.

### 4.1 Outcomes

#### 4.1.1 Impact of hospital walking on length of stay

**FIGURE 2:**
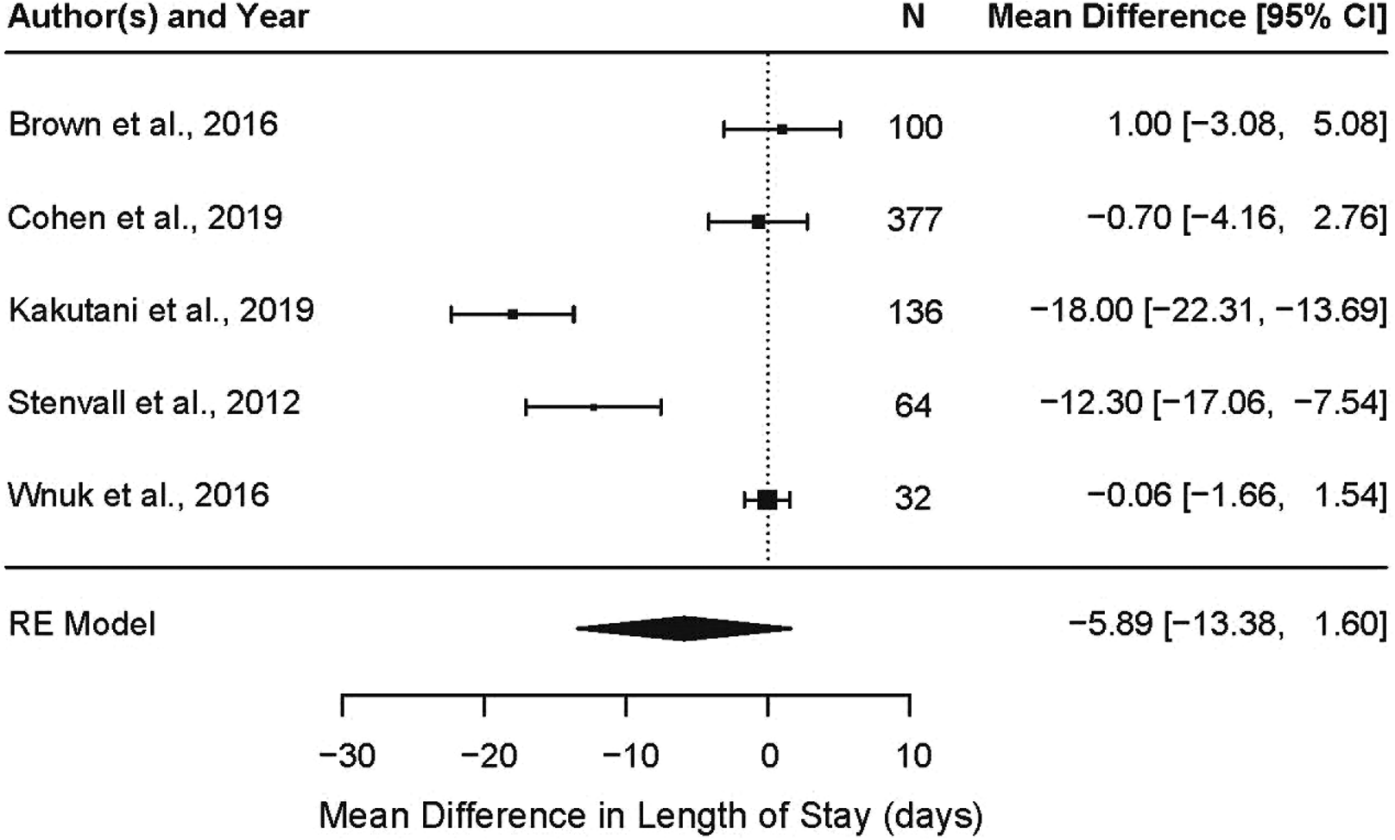
Forest plot of mean difference in length of stay (LOS) between intervention and control groups among experimental studies reporting LOS as mean/SD

Seven studies reporting hospital LOS were used in meta‐analysis. Figure 2 illustrates the difference between groups in mean LOS in days across all intervention studies that reported LOS as mean ± SD. Random effects meta‐analysis showed that combined average difference in LOS across studies was −5.89 days with an observed significance level of 0.1233. Since Stenvall et al. (2012) focused on patients with dementia specifically, making this study unique from the other studies, we completed a sensitivity analysis without this study data and found no meaningful difference in the combined effect (data not shown).

Figure S3 illustrates the difference between groups in mean LOS in days when one intervention study (Hastings et al., 2014) reporting LOS as median/IQR was added to the random effects model. The combined average effect size across studies was −4.94 days with an observed significance level of 0.1284. Addition of one observational study (Fisher et al., 2016) reporting mean difference between inpatients who were not re‐hospitalized (walked more during the index hospitalization) compared with those that were re‐hospitalized (walked less during the index hospitalization) in the random effects model of the intervention studies reporting mean LOS identified a combined average effect size of −5.13 with an observed significance level of 0.1082 (Figure S4). Across multiple scenarios, meta‐analysis identified no significant effect of hospital walking on LOS.

Two intervention studies (Chen et al., 2017; Ortiz‐Alonso et al., 2020) also assessed LOS changes following a hospital walking program, but the reported data could not be used in meta‐analysis. While Chen et al. identified a significant reduction in LOS with hospital walking (median 12 days vs. 14 days for control; *p* = .04), Ortiz‐Alonso et al. showed no difference between groups (median 6 days vs. 7 days for control; *p* = .25).

#### 4.1.2 Hospital walking dose

The secondary aim of this review was to identify and characterize hospital walking dose among older inpatients. Walking dose in steps or distance units is often measured with objective tools that give longitudinal monitoring and comparative analysis across studies. Six studies were identified that reported walking dose or distance as steps, feet or meters per 24 hr during hospitalization. The most common method of reporting walking was in steps/24 hr (Table 1).

In the USA, two observational studies by showed that older patients walk ˂1,000 steps/24 hr, and oftentimes much less than 1,000 steps based on Q1 and Q3 data represented in Table 1 (Fisher et al., 2011; Fisher et al., 2016). The patients enrolled in the studies by Fisher et al. were commonly admitted to acute care for cardiopulmonary reasons and about one‐third were dependent in activities of daily living (ADL) prior to or during the studies. Alternatively, an observational study in Japan identified that older patients admitted to acute cardiac care walk between 2,210–4,309 steps/24 hr (Izawa et al., 2015). Patients enrolled in this study were only admitted for cardiovascular reasons. Uniquely, this study examined differences in hospital walking amount based on biological sex and identified that male cardiac inpatients took significantly more steps/24 hr than older female cardiac inpatients. This study also identified that the average daily number of steps taken in the hospital is positively correlated to gait speed. In Israel, an experimental study was executed that focused on enhancing mobility of inpatients by reducing barriers to mobility (Cohen et al., 2019). The study by Cohen et al. reported steps/24 hr among patients in the control and walking groups and identified that the control group patients took on average 1791 steps, while walking group patients took on average 3,205 steps. This study also uncovered that increased hospital walking significantly lowered odds of experiencing a decline in ADL compared with the control group. Finally, other studies, including one from Australia and another from America showed that walking group patients increased walking amount throughout hospitalization to a greater extent than control group patients (Adogwa et al., 2017; Killey & Watt, 2006). Uniquely, the American study by Adogwa et al. identified that early mobilization among surgical patients resulted in increased walking distance during hospitalization compared with controls (Adogwa et al., 2017; Cohen et al., 2019; Killey & Watt, 2006).

#### 4.1.3 Study quality assessment

Quality bias analysis identified that included studies rated as moderate or low for risk of bias. Among the RCTs using the RoB2 tool (Sterne et al., 2019), 3 studies indicated loss to follow up (missing outcomes data) during the study and 1 study did not report loss to follow up (Figure S1). It was also determined using the Jadad Scale (Jadad et al., 1996) that 4/6 RCTs (Brown et al., 2016; Chen et al., 2017; Ortiz‐Alonso et al., 2020; Stenvall et al., 2012; Valenzuela et al., 2020) did not use assessor masking or did not report using assessor masking which could have also contributed to study bias. Quality bias appraisal of non‐randomized intervention studies using the ROBINS‐I tool (Figure S2) identified that 2 studies reported participant selection procedures that could have contributed to bias and 2 studies did not report how participants were selected (Sterne et al., 2016). Missing outcomes data was also a source of bias for 1 study while 2 studies did not report on missing data. Quality bias analysis for the observational studies using the ROBINS‐E tool (Higgins, Morgan, et al., 2022; Higgins, Thomas, et al., 2022) also identified 2 studies that did not report missing outcomes data, which could be a source of bias (Table S2).

**FIGURE 3:**
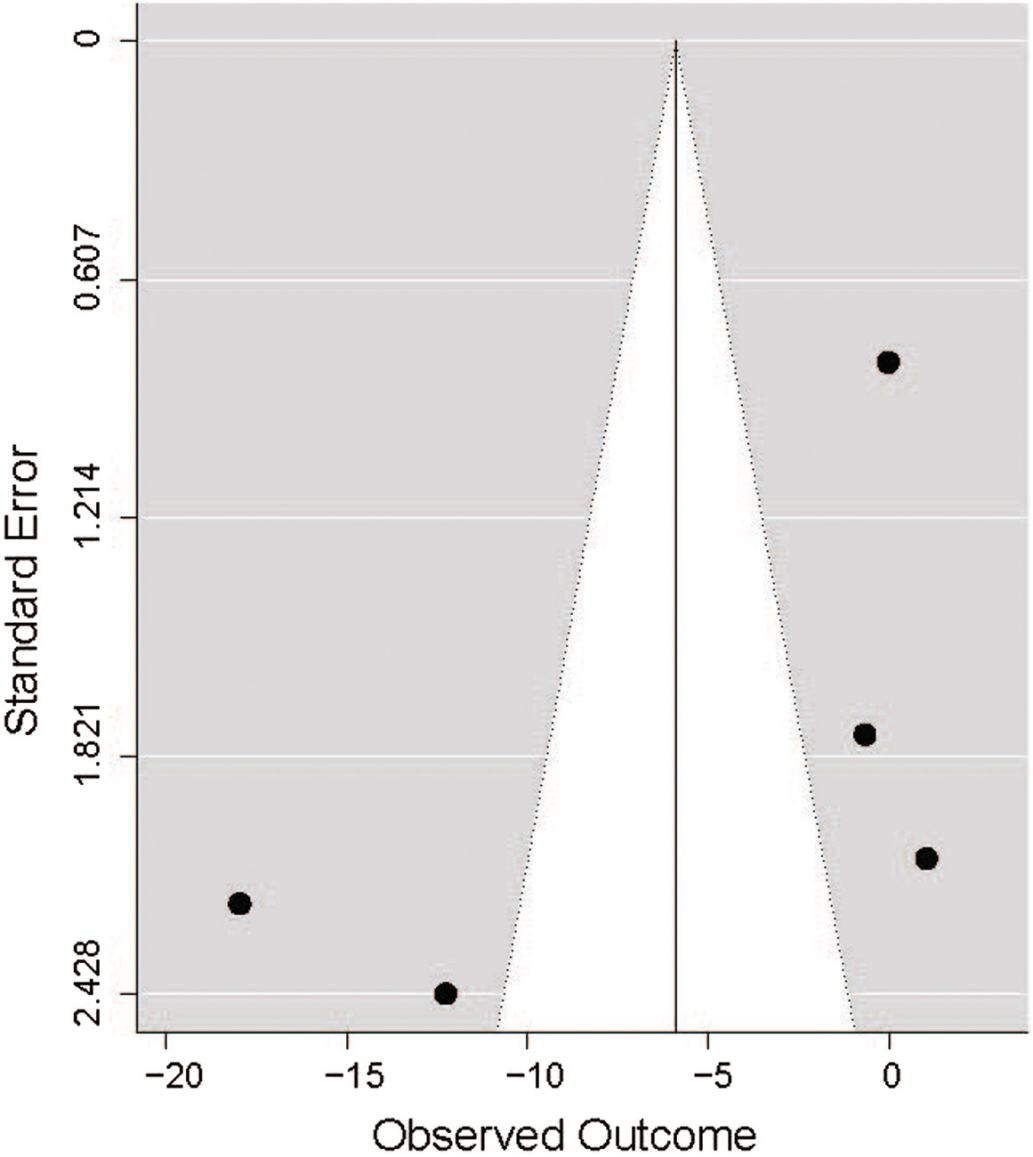
Funnel plot of estimates (estimated difference in mean length of stay, LOS) vs. precision (standard effort) of experimental studies reporting LOS as mean/SD

Funnel plots for difference in mean LOS showed considerable heterogeneity (Figure 3, Figures S5 and S6). Cochran's *Q* test revealed that across experimental studies reporting mean LOS *Q* = 78.72, *df* = 5, *p* < .0001. The heterogeneity *I* ^2^ statistic was 96%. Cochran's Q test across experimental studies plus one study reporting the difference in LOS as median/IQR showed that *Q* = 79.73, *df* = 6, *p* < .0001. The heterogeneity *I* ^2^ statistic was 95%. Finally, Cochran's *Q* test across experimental studies reporting mean LOS plus one observational study reporting mean LOS identified that *Q* = 78.98, *df* = 6, *p* < .0001. The heterogeneity *I* ^2^ statistic was 95%. Almost all studies showed low precision in their estimates. None of the studies were in the expected ratio between the magnitude of the measured outcome and the measured precision, which suggests a potential publication bias with omission of higher quality, possibly negative trials. Collectively, these measures raise concerns about the quality of trial reporting (Macaskill et al., 2001). The fail‐safe N could not be calculated as there was not a statistically significant impact of hospital walking on LOS across studies.

## 5 DISCUSSION

This systematic review aimed to identify all studies investigating the effect of hospital walking for older acute care and surgical inpatients and those reporting walking dose in this population. Results of the review identified that hospital LOS was the most common outcome measured in studies testing a hospital walking program. Meta‐analysis of the difference in mean LOS across studies determined that hospital walking does not result in a statistically significant reduction in LOS for patients who are ≥65 years. Heterogeneity between studies based on the random effects model was considerable. It was also identified that hospital walking dose has not been widely investigated among older patients and that steps/24 hr is the most common measurement method.

It is understood that low mobility during hospitalization is a critical event for older patients placing them at risk for loss of independence in mobility and function and for chronic disability (Zisberg et al., 2011; Brown et al., 2004; Gill et al., 2004; Gill et al., 2010). Alternatively, increased mobility during hospitalization has been shown to prevent loss of functional independence (Brown et al., 2016; Cohen et al., 2019; Kakutani et al., 2019; Yohannes & Connolly, 2003). As a result, the American Geriatrics Society (AGS) recently published a White Paper suggesting that hospital mobility should be measured and promoted among older patients (Wald et al., 2019). The AGS task force described mobility as a clinical indicator or standard of care that is not widely assessed or encouraged in acute care hospitals (Wald et al., 2019). In light of findings uncovered in the present review that few randomized controlled trials have tested hospital walking programs specifically in older hospitalized adults and that studies that have been completed are inconsistent in reported findings, it is possible that generating additional supporting evidence across multiple well‐designed high‐quality trials would help to stimulate hospital adoption of regular mobility assessment and promotion.

Even though most studies (6/7) in this review showed that inpatient walking tended to or significantly reduced LOS, random effects analysis of the data across studies did not translate into a significant reduction overall. These findings are in contrast to those presented in a review by Cortes et al. (2019) which showed a statistically significant decline in LOS among adult patients who mobilized during hospitalization. Importantly, while our investigation focused solely on studies involving older adults and walking interventions, the review by Cortes et al. had a broader definition of mobility encompassing ambulation and exercise and balance‐promoting activities and included adults ≥18 years. These design differences likely contributed to the incongruence of our findings. Additionally, Cortes et al. reported moderate heterogeneity across studies for differences in LOS (*I* ^2^ statistic ~58%), which is much lower than the large heterogeneity discovered in our random effects model (*I* ^2^ statistic ~95%–96%). Their focus on randomized controlled trials might have contributed to this difference which supports that higher‐quality design of hospital walking studies for older inpatients could potentially improve consistency of findings across studies.

Alternatively, hospital LOS might not be the most effective metric for judging benefit of hospital walking for older inpatients. LOS is the product of multiple factors and not simply a function of the patient or disease. As described in a recent scoping review, hospital LOS is affected by 3 groups of variables: healthcare systems characteristics, social and family characteristics, and patient characteristics (Buttigieg et al., 2018). For example, a study showed that the pre‐admission and postdischarge community healthcare environment impacts LOS for older patients with heart failure (Wright et al., 2003). Another study showed that the availability of hospital beds and being admitted from the emergency department can also affect LOS (Sun et al., 2013). Since meta‐analyses are considered the highest level of evidence and vital for developing evidence‐based practice in healthcare, future studies should focus on measuring outcomes that are closely related to older patient health and well‐being and allow comparison across studies completed in diverse geographical locations, communities, and healthcare systems.

Other outcomes to consider measuring routinely in future hospital‐based research among older patients are gait speed and in‐hospital falls. Gait speed has been described as a clinically relevant indicator of functional independence and life expectancy for older adults (Ostir et al., 2012; Studenski et al., 2011), and it is sensitive to the negative effects of hospitalization. One study showed that hospitalization is associated with gait speed decline among older patients who were functionally independent in ADL and walking ability at hospital admission (Duan‐Porter et al., 2019). Further, gait speed has been used as an outcome in an intervention trial targeting frailty among community‐dwelling older adults (Fairhall et al., 2008). Yet, despite being a rapid, inexpensive, and reliable in measuring physical function (Cesari et al., 2005; Guralnik et al., 2000), only one observational study of our review examined gait speed in relation to hospital walking. Izawa et al. (2015) identified a positive correlation between average daily number of steps taken in‐hospital and gait speed, *r* = .46, *p* < .001.

Another metric to consider is in‐hospital falls. Evidence support that hospitalized adults are at risk of falling which can lead to fractures, soft tissue injuries and psychological distress (Oliver et al., 2010; Schwendimann et al., 2008). Hospital walking programs may decrease risk of falls among older inpatients by combating the negative physiologic sequela that occurs from imposed bed rest (Creditor, 1993). Our review identified 4 intervention studies that assessed falls during hospitalization. Two studies found no difference in number of falls between the intervention and control groups (Cohen et al., 2019; Hastings et al., 2014) and 2 studies described falls occurring among control group patients but not walking group patients (Brown et al., 2016; Killey & Watt, 2006). More studies are needed to complete meta‐analysis across studies to determine whether walking programs reduce in‐hospital falls for older patients.

With a growing understanding of the importance of hospital mobility over the last few decades and improved methods for measuring mobility objectively, we hypothesized that studies reporting mobility dose or distance have also increased. Interestingly, we only identified 6 studies reporting hospital walking dose as steps/24 hr or distance/24 hr. Qualitative analysis of studies reporting steps/24 hr identified that those based in the USA reported older inpatients walked on average ~ 600–700 steps/24 hr (Fisher et al., 2011; Fisher et al., 2016). This is in line with another study completed in a California hospital showing that among all adult patients who are ≥65 years walked on average ˂1,000 steps/24 hr (~900 steps; Sallis et al., 2015). Studies completed in Japanese and Israeli hospitals reported patients walked on average ˃1,000 steps/24 hr (Cohen et al., 2019; Izawa et al., 2015). While it is unclear why mobility dose is different across countries, it is possible that known and unknown differences of the patient samples used is involved and differences in study design.

This study has notable strengths. First, to the best of our knowledge, this is the first systematic review and meta‐analysis conducted examining the impact of hospital‐based walking programs for older inpatients. Second, this investigation was not restricted in timeframe, geography, or research design which allowed a comprehensive analysis on the research and publication landscape related to hospital mobility and older inpatients. Along the same lines, inclusion of studies from a variety of geographical areas and populations without limitation on when the study was completed increases the generalizability of the findings.

An important limitation of the investigation is the inability to use data from all included studies in meta‐analysis. Intervention studies that could not be included but reported hospital LOS either did not include a control group or did not report quartile values for the IQR. Further, the heterogeneity between included studies which could be a result of differences in study design or clinical populations make combining data from individual studies less reliable statistically. However, the observed heterogeneity illustrates the need for more uniformity among studies in the future. Finally, the time‐period of this systematic review focused on literature published before December 2020. It is possible additional studies have been published since our systematic search of the electronic databases that meet our eligibility criteria. A non‐exhaustive search of PubMed for studies published between December 2020 and December 2021 identified two additional studies. An RCT on a geriatric ward of an Italian hospital tested a hospital walking intervention among older patients and measured LOS as a secondary outcome (Gazineo et al., 2021), and an experimental study on a geriatric ward of an Israeli hospital assessed a walking program among older hospitalized patients with dementia and measured LOS (Oliven et al., 2021). Neither study showed a significant reduction in hospital LOS with increased mobility during hospitalization. These findings are consistent with the findings of this review.

## 6 CONCLUSION

This review identified that LOS is a common metric for walking intervention impact, and yet it might not be useful for illustrating benefit of hospital walking among older inpatients. Individual studies show benefits of walking programs for older patients, meta‐analysis across studies failed to show a statistically significant decrease in LOS with increased walking during hospitalization. Further, we report important heterogeneity across studies supporting the need for additional high‐quality research focused on hospital walking and impact on outcomes related to physical function of the older inpatient. Finally, this review identified that only 6 studies have reported dose of hospital walking and that steps or distance/24 hr is commonly measured. Walking dose reported appears to vary across country, however contributing factors to differences in hospital walking dose remain unknown.

## AUTHOR CONTRIBUTIONS

Made substantial contributions to conception and design, or acquisition of data, or analysis and interpretation of data: CL, YZ, TW, JB, EH, JG, SO, LR, SGM, CCL, CHS, REK, BK, CJB; Involved in drafting the manuscript or revising it critically for important intellectual content: CL, YZ, REK, BK, CJB. Given final approval of the version to be published. Each author should have participated sufficiently in the work to take public responsibility for appropriate portions of the content; CL, YZ, TW, JB, EH, JG, SO, LR, SGM, CCL, CHS, REK, BK, CJB. Agreed to be accountable for all aspects of the work in ensuring that questions related to the accuracy or integrity of any part of the work are appropriately investigated and resolved: CL, YZ, TW, JB, EH, JG, SO, LR, SGM, CCL, CHS, REK, BK, CJB.

All authors have agreed on the final version and meet at least one of the following criteria [recommended by the ICMJE (http://www.icmje.org/recommendations/)]:

- substantial contributions to conception and design, acquisition of data or analysis and interpretation of data;
- drafting the article or revising it critically for important intellectual content.

## FUNDING INFORMATION

Research reported in this manuscript was supported by the National Center for Advancing Translational Sciences of the National Institutes of Health under award number UL1TR003096. The content is solely the responsibility of the authors and does not necessarily represent the official views of the NIH.

## CONFLICT OF INTEREST

The authors have no conflicts of interest to declare.

## Supporting information


Figure S1

Figure S2

Figure S3

Figure S4

Figure S5

Figure S6

Table S1

Table S2

## Data Availability

The data that support the findings of this study are available from the corresponding author upon reasonable request.
