# Supplementary figures and images for: A systematic review and meta‐analysis: Assessment of hospital walking programs among older patients
Source: Nurs Open. 2022 Nov 28;10(4):1942–53. doi: 10.1002/nop2.1496 (PMC10006621; doi:10.1002/nop2.1496)

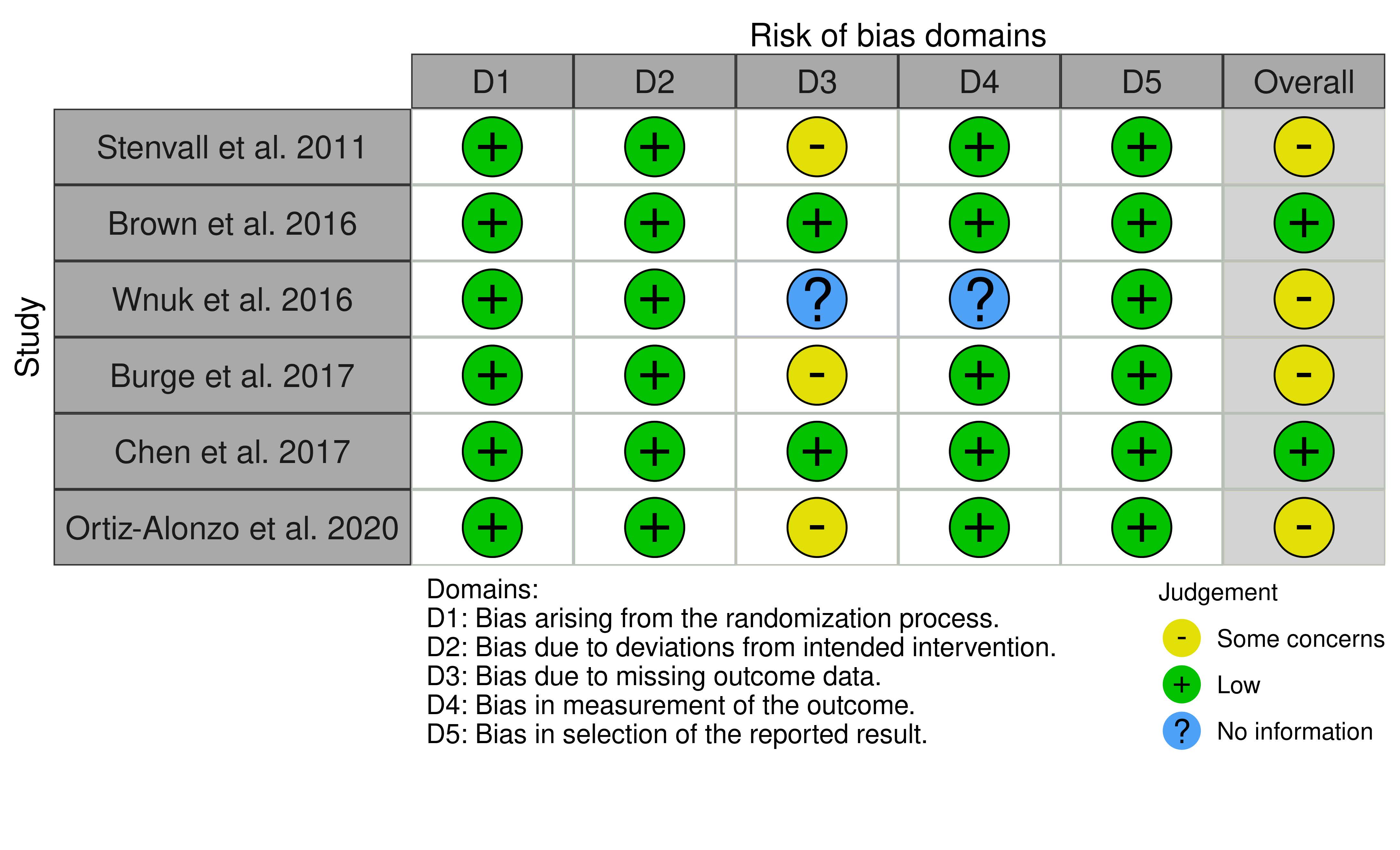

Supplement: Supplementary file 1 — Figure S1 [file NOP2-10-1942-s002.jpg]

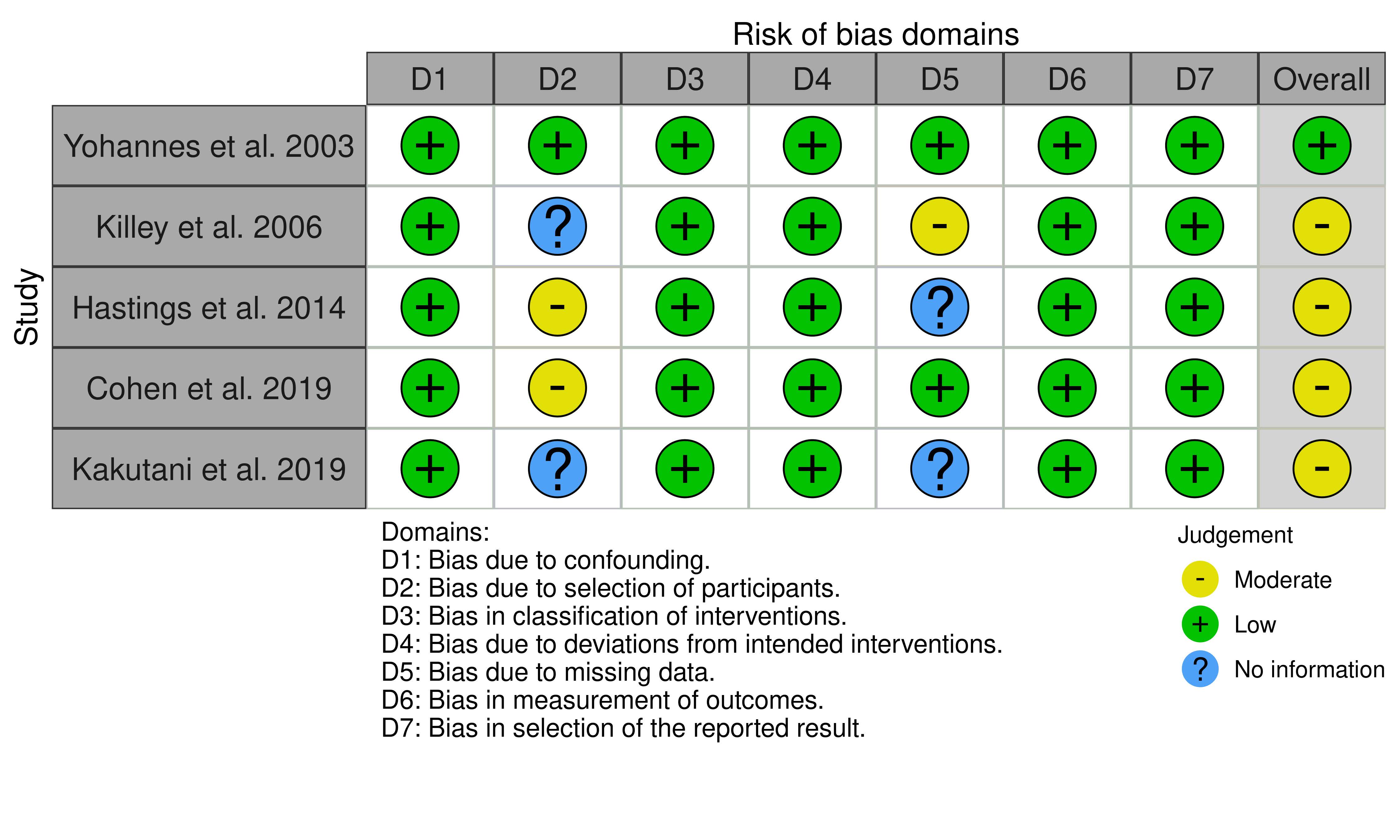

Supplement: Supplementary file 2 — Figure S2 [file NOP2-10-1942-s008.jpg]

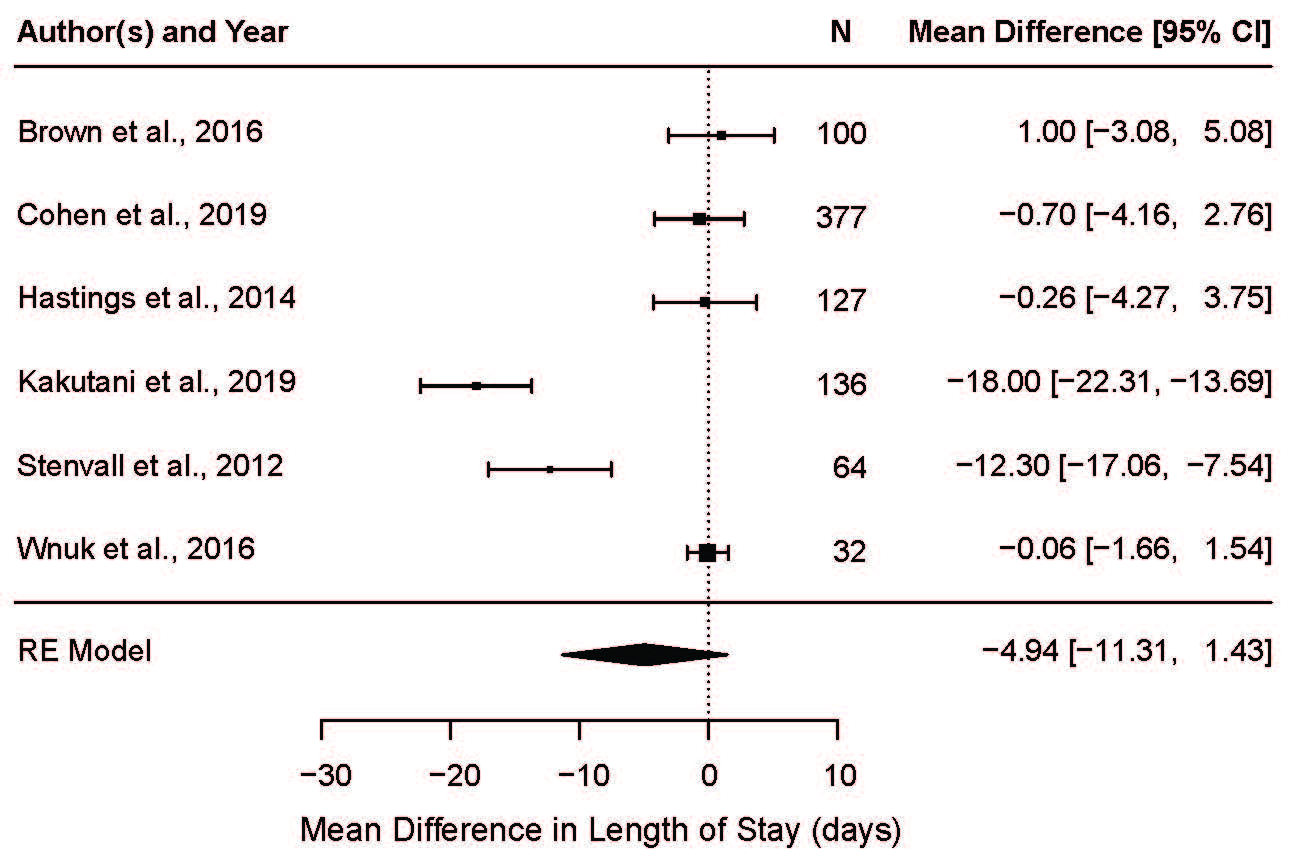

Supplement: Supplementary file 3 — Figure S3 [file NOP2-10-1942-s005.jpg]

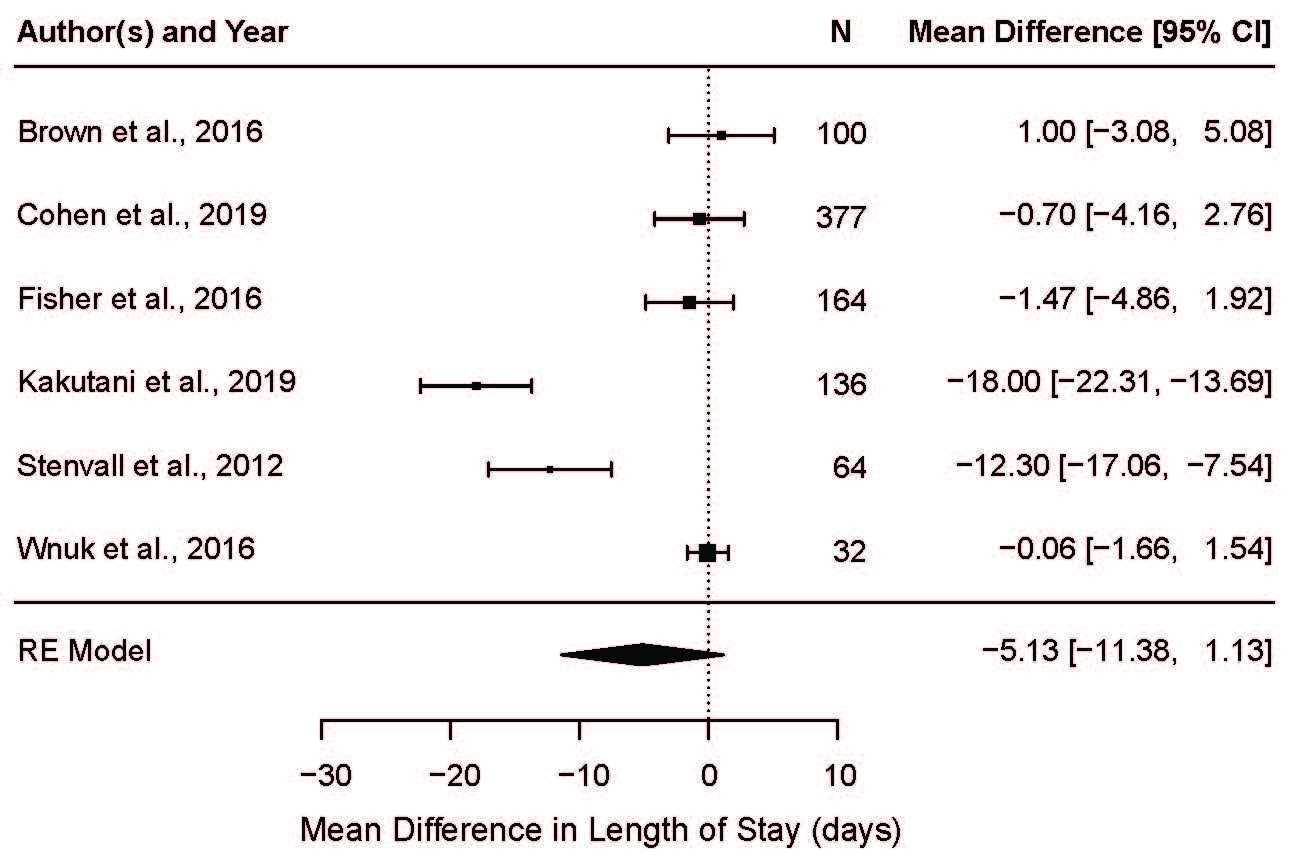

Supplement: Supplementary file 4 — Figure S4 [file NOP2-10-1942-s007.jpg]

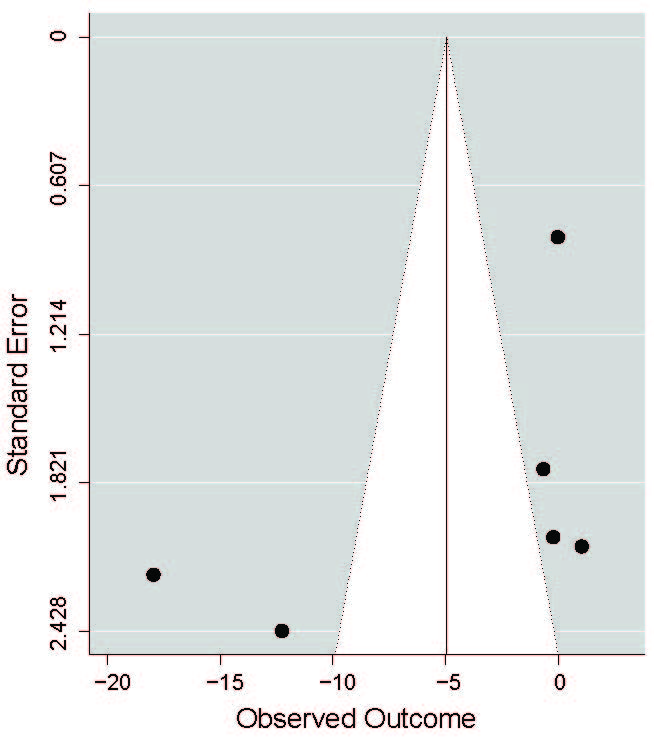

Supplement: Supplementary file 5 — Figure S5 [file NOP2-10-1942-s001.jpg]

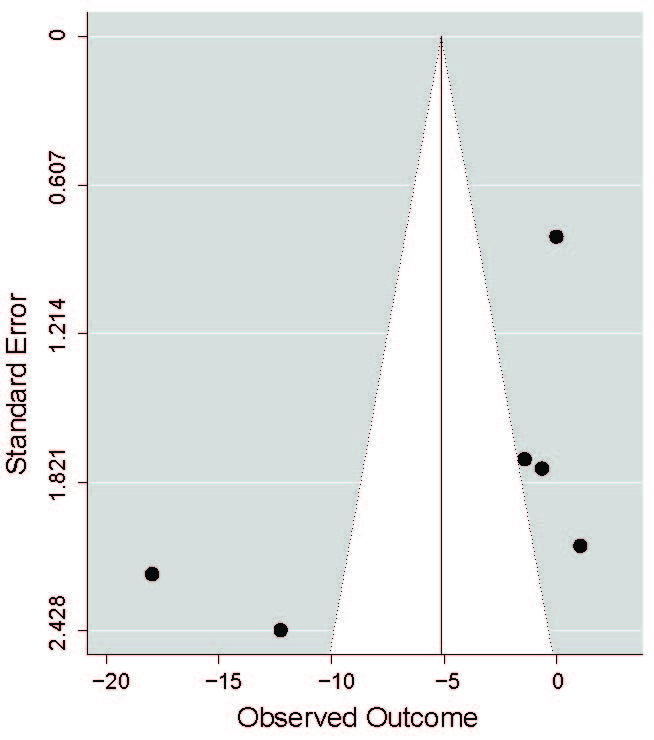

Supplement: Supplementary file 6 — Figure S6 [file NOP2-10-1942-s003.jpg]

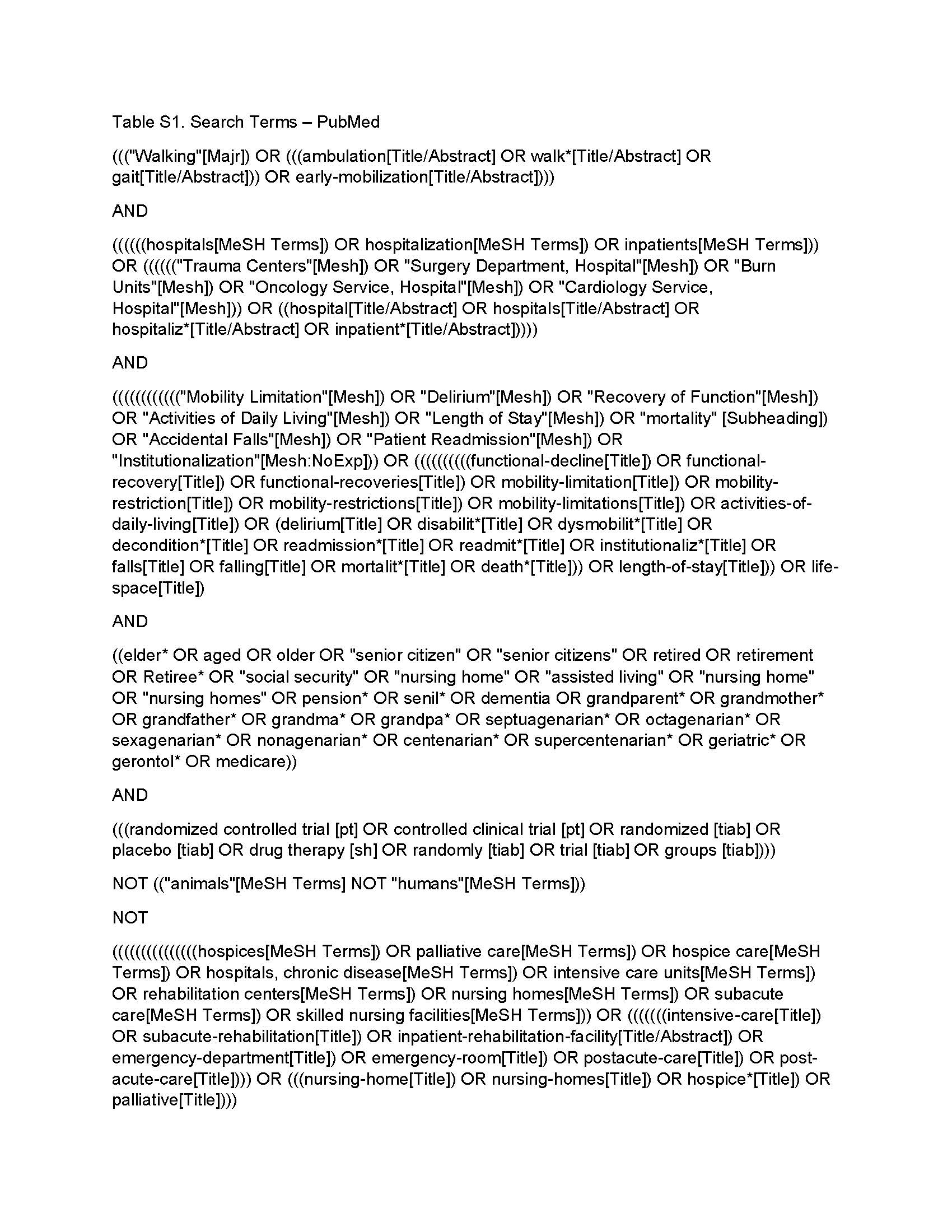

Supplement: Supplementary file 7 — Table S1 [file NOP2-10-1942-s004.jpg]

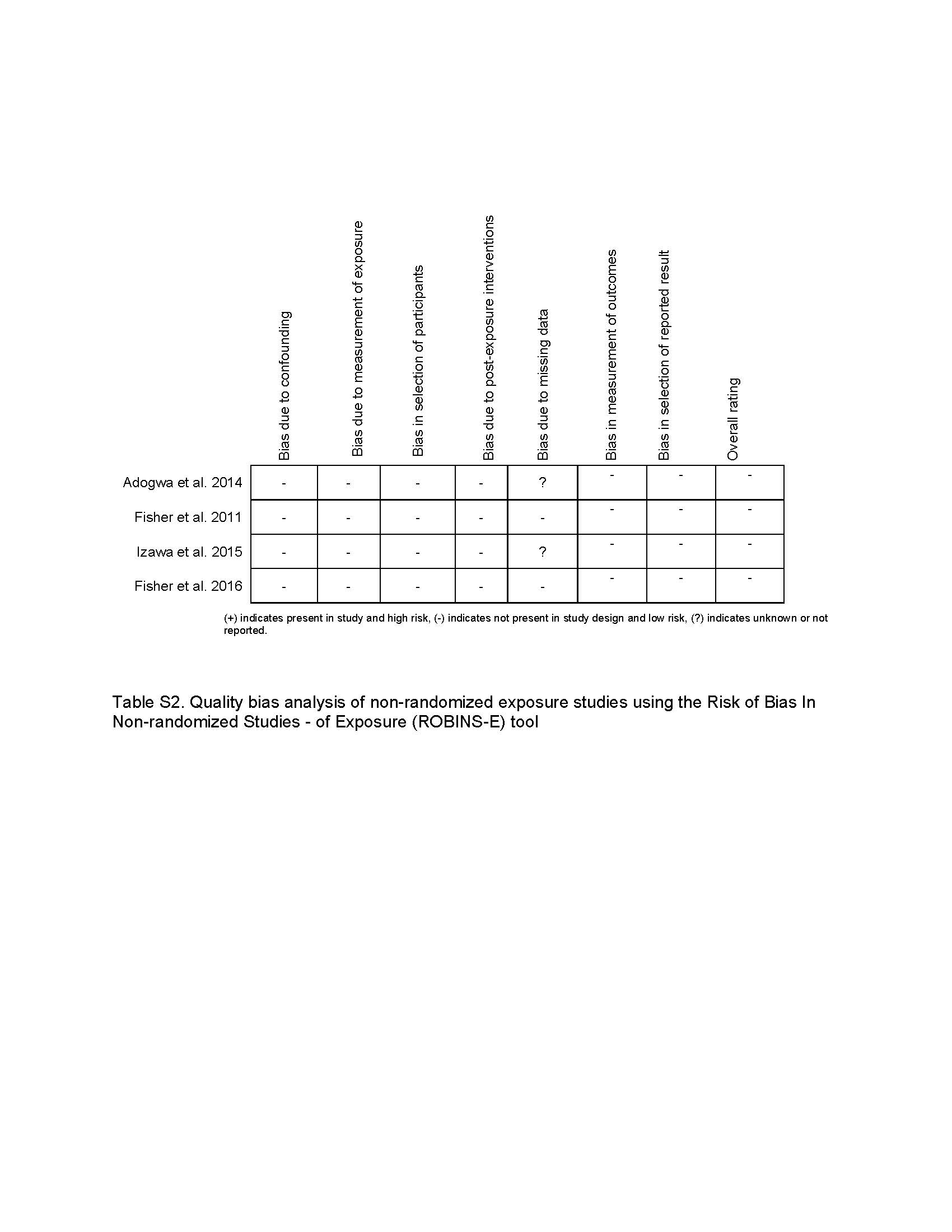

Supplement: Supplementary file 8 — Table S2 [file NOP2-10-1942-s006.jpg]
